# Psychological distress, health-promoting lifestyle and sociodemographic factors in Honduran university students: a structural equation model

**DOI:** 10.1093/heapro/daae082

**Published:** 2024-07-18

**Authors:** Marcio Alexander Castillo-Díaz, María Candelaria Martínez, Carlos Alberto Henao Periañez, Dilcia Sauceda-Acosta

**Affiliations:** Orientation and Student Affairs Departament (VOAE) and Faculty of Social Sciences, Universidad Nacional Autónoma de Honduras (UNAH), Ciudad Universitaria, Boulevard Suyapa, Tegucigalpa 11101, Honduras; Orientation and Student Affairs Department (VOAE), Universidad Nacional Autónoma de Honduras (UNAH), Ciudad Universitaria, Boulevard Suyapa, Tegucigalpa 11101, Honduras; School of Nursing, Faculty of Health, Universidad del Valle, Sede San Fernando, 4b 36-00 St., Cali 760042, Colombia; Graduate Program in Epidemiology, Research Institute in Medical Sciences and Right to Health, Faculty of Medical Sciences, Universidad Nacional Autónoma de Honduras (UNAH), Ciudad Universitaria, Boulevard Suyapa, Tegucigalpa 11101, Honduras

**Keywords:** mental health, health behavior, university students, social determinants of health, observational study, structural equation modeling

## Abstract

This study sought to analyze an explanatory model on the relationship among sociodemographic factors, health-promoting lifestyle behaviors and psychological distress (depression, anxiety and stress) in college students. This is an observational, analytical and cross-sectional study conducted on a national sample of 4203 students who entered a macro university in Honduras in 2021, 2022 and 2023. We used a sociodemographic survey, the Health-Promoting Lifestyle Profile (HPLP-II) and the Depression, Anxiety and Stress Scales (DASS-21). Univariate analysis and a multivariate structural equation model were conducted. The average HPLP-II score was 117.45 (± 23.41), and the average DASS-21 score was 20.06 (± 14.16). The multivariate model showed a good data fit (comparative fit index = 0.951; Tucker–Lewis index = 0.957; root mean square error of approximation = 0.067 [90% CI = 0.067–0.068]). Results indicate that being a woman (β = 0.11; *p* < 0.001) and being enrolled in biological and health sciences (β = 0.09; *p* < 0.001) significantly predict HPLP-II scores. Furthermore, being a woman (β = 0.17; *p* < 0.001), age (β = 0.10; *p* < 0.001) and having pre-existing medical conditions (β = 0.16; *p* < 0.001) significantly explain part of the variance of DASS-21. A significant reverse relationship between health-promoting behavior and psychological distress was shown (*r* = −0.36; *p* < 0.001). This study identifies protective and risky sociodemographic factors linked to health-promoting lifestyle behaviors and psychological distress. Our findings have implications for developing comprehensive intervention policies and strategies to promote health in higher education settings.

**Contribution to Health Promotion**

- Sociodemographic factors were linked to health-promoting lifestyles and psychological distress.
- Gender and enrollment in health sciences predict health-promoting lifestyles.
- Gender, age and pre-existing medical conditions predict psychological distress.
- Health-promoting lifestyles and psychological distress were negatively correlated.
- Findings may assist the implementation of university health promotion strategies, identifying risk groups.

## BACKGROUND

Higher education not only fosters intellectual and professional growth but also has substantial effects on an individual’s biological, psychological and social well-being (Liu *et al*., 2019; Douwes *et al*., 2023; Chaudhry *et al*., 2024). The commencement of university entails the necessity for students to adapt to novel academic requirements, institutional structures, time management adjustments, social interactions, familial relationships and the development of self-regulation abilities in their role as students (Bewick *et al*., 2010; Liu *et al*., 2019; Castillo-Díaz *et al*., 2022).

The transition from secondary to tertiary education is seen as a crucial phase for the development of behaviors that can impact students’ health and well-being (Worsley *et al*., 2021). Health-promoting behaviors refer to actions undertaken by individuals to enhance their emotional, physical and spiritual well-being (Fernandez *et al*., 2019). These behaviors have a broad impact on the physical and psychological well-being of students (Amiri *et al*., 2019; Al-Momani, 2021). University students are susceptible to adopting unhealthy behaviors and exhibiting signs of psychological distress (Almutairi *et al*., 2018; Diaz-Godiño *et al*., 2019). Furthermore, studies have demonstrated statistically significant correlations between healthy lifestyles and mental health (Saneei *et al*., 2016; Davarinejad *et al*., 2020; Safaie *et al*., 2020; Tang *et al*., 2021; Dash *et al*., 2022).

Recent research indicates a correlation between sociodemographic factors and the adoption of healthy behaviors among university students (Wang and Geng, 2019). Gender disparities exist in the profiles of health-promoting behaviors, as men exhibit higher levels of physical exercise and superior stress management skills (Ulla Diez and Perez-Fortis, 2010; Alzahrani *et al*., 2019; Ghanim *et al*., 2021). Conversely, women obtain higher results in approaching interpersonal relationships (Ghanim *et al*., 2021). In addition, women report a greater food intake during times of stress, whereas men report a lower consumption of fruits and vegetables (Müller *et al*., 2022).

Research has also examined the correlation between socioeconomic status and health-promoting behaviors, suggesting that students from higher-income families exhibit superior health-promoting behaviors (Ulla Diez and Perez-Fortis, 2010; Alzahrani *et al*., 2019). Overall, socioeconomic status has a substantial impact on individuals’ physical health (Wang and Geng, 2019).

On the other hand, literature shows correlations between sociodemographic variables and the prevalence of anxiety, depression and stress. Adolescents and young adults are more prone to experiencing depression and anxiety disorders (Ramón-Arbués *et al*., 2020; Li *et al*., 2022). Evidence from systematic reviews indicates a higher prevalence of depression among university students compared to the general population (Ibrahim *et al*., 2013), with ~ 25% of undergraduates in low- and middle-income countries experiencing symptoms of depression (Akhtar *et al*., 2020). Similarly, research indicates that women exhibit higher levels of depression and anxiety, particularly during the initial years of their university education (Tesfahunegn and Gebremariam, 2019; Gao *et al*., 2020). Furthermore, it is noteworthy that health sciences students show a higher prevalence of psychological disorders in comparison to students in other academic fields (Ngasa *et al*., 2017). Regarding economic status, research demonstrates that students with low family income, low social support and a family history of mental disorders have a higher prevalence of depression, anxiety and stress (Tesfahunegn and Gebremariam, 2019).

To the best of our knowledge, there is a scarcity of studies that address the relationship among sociodemographic factors, health-promoting behaviors and psychological distress in Latin American higher education settings. In recent literature, there is evidence from studies in Peru (Diaz-Godiño *et al*., 2019), Mexico (Ulla Diez and Perez-Fortis, 2010) and Colombia (Zambrano Bermeo *et al*., 2023). Nevertheless, within a unique multivariate model, these studies fail to incorporate simultaneous associations and predictions of sociodemographic variables on health-promoting behaviors and psychological distress. This integration may enable a more robust approach to the complex interconnection of the variables.

By characterizing sociodemographic factors that are predictive of healthy lifestyles and psychological distress, it is feasible to identify priority groups at risk and develop intervention programs that may be more effective (Nho and Chae, 2021). Promoting healthy lifestyles and mental health is a crucial priority in public health, encompassing educational policies and the development of initiatives to ensure the overall well-being of the university student community.

**Fig. 1:**
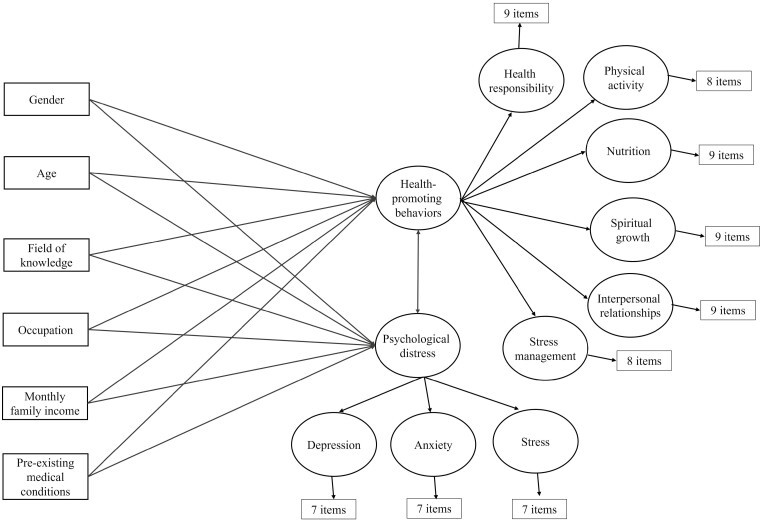
Proposed theoretical model.

This study aims to analyze an explanatory model on the relationship among sociodemographic factors, health-promoting lifestyle and psychological distress (depression, anxiety and stress) in college students. According to this model, factors such as gender, age, area of knowledge, occupation, family income and pre-existing medical conditions may predict health-promoting behavior and psychological distress. In addition, it is hypothesized that there is an inverse relationship between the two outcome variables (Figure 1).

## METHODS

This is an observational and analytical cross-sectional study that was conducted among a higher education population of a macro university in Honduras.

### Study settings and participants

The population consisted of 27 759 students entering university during the years 2021, 2022 and 2023. The study included undergraduate students who were at least 18 years old and were enrolled in various academic programs and campuses of the major universities of Honduras. The minimum sample size required was computed based on the suggested structural equation model, which consists of 79 observable variables (6 sociodemographic and 73 items) and 11 latent variables, as shown in Figure 1. A minimum of 900 participants was deemed sufficient to identify effect sizes (correlations and betas) of at least 0.15, with a significance level of α = 0.05 and a statistical power of 1 − β = 0.80 (Soper, 2023). The participants in this study were chosen via intentional, non-probabilistic sampling.

### Measurements

#### Sociodemographic characteristics

The sociodemographic data were gathered using a screening questionnaire administered to first-year students. For this study, we considered gender, age, field of knowledge, monthly family income, current occupation and pre-existing medical conditions. Gender was categorized into three groups: women, men and other/non-binary. We used gender as an inclusive term, this approach acknowledges gender as a social construct and a social identity self-reported by the participants (Heidari *et al*., 2016). Age was stratified into four groups: < 20, 20–24, 25–29 and ≥ 30 years. We classified the student’s areas of knowledge into four overarching groups: biological and health sciences, economic sciences, engineering/physics-mathematics and social sciences/humanities. The levels of monthly family income were defined based on the average minimum wages in Honduras during the study’s timeframe (Ministry of Labor and Social Security—Honduras, 2024). This variable was categorized as follows: low level (equivalent to a limit of one minimum wage, ~ 11 400 lempiras), medium level (from more than one minimum wage to four minimum wages [11 401–45 600 lempiras]) and high level (more than four minimum wages [>45 600 lempiras]). The exchange rate for the US dollar was around 24.50 lempiras at the time of the study (Central Bank of Honduras, 2023). The approximate amount of monthly family income was self-reported by students. Current occupation was categorized as just studying and studying/working. Self-reported pre-existing medical conditions were assessed into yes and no categories.

#### Health-Promoting Lifestyle Profile (HPLP-II)

This instrument assesses health-promoting behaviors using a Likert scale with 52 items. Each item has four response alternatives, ranging from never to routinely (Walker *et al*., 1987). HPLP-II comprises six factors: health responsibility (nine items), physical activity (eight items), nutrition (nine items), spiritual growth (nine items), interpersonal relationships (nine items) and stress management (eight items). Higher scores of each factor indicate stronger adherence to practices that foster healthy lifestyles. Literature provides evidence of the psychometric features of the instrument, specifically its validity and reliability, among university students (Davis and De Guzman, 2022; Enríquez-Reyna *et al*., 2022). In this study, a hierarchical measurement model was tested, composed of six first-order factors and a general second-order factor, called health-promoting behavior. A confirmatory factor analysis of items indicated a good fit of the model (*X*^2^ = 28 741.17; *df* = 1268; *p* < 0.001; comparative fit index [CFI] = 0.961; root mean square error of approximation [RMSEA] = 0.072 [90% CI = 0.071–0.073]), with all factor loadings above 0.431. Reliability in the current sample shows adequate omega indices (Ω) for each of the subscales (health responsibility = 0.86; physical activity = 0.89; nutrition = 0.78; spiritual growth = 0.88; interpersonal relationships = 0.79; stress management = 0.77), and a second-order omega (Ω_ho_) of 0.90 for the general factor.

#### Depression, Anxiety and Stress Scales (DASS-21)

Instrument widely used internationally to evaluate psychological distress in both the clinical and general population (Lovibond and Lovibond, 1995). The test contains 21 items divided into three subscales: depression, anxiety and stress (seven items, respectively). Each item uses a four-point Likert scale to assess what the person encountered or felt in the past week. DASS-21 has substantial evidence supporting its psychometric features (Lee *et al*., 2019; Zanon *et al*., 2020). In this study, we used a hierarchical measuring model, consisting of three distinct factors and a second-order general factor, referred to as psychological distress (Makara-Studzińska *et al*., 2022). The structural validity of the model, tested through confirmatory analysis of items, indicated a good fit (*X*^2^ = 1318.44; *df* = 186; *p* < 0.001; CFI = 0.997; RMSEA = 0.043 [90% CI = 0.041–0.045]), with all factor loadings above 0.519. The depression, anxiety and stress subscales showed Ω values of 0.91, 0.87 and 0.88, respectively. The general factor (i.e. psychological distress) indicated an Ω_ho_ of 0.93.

### Procedures and ethics

The process of data collection was conducted electronically via an institutional email-distributed form. The collection took place from January to February in 2021, 2022 and 2023. The data from this study are component of a broader multicenter research project conducted in collaboration between Honduras and Colombia titled ‘Adoption of preventive behaviors against COVID-19 among university students in Honduras and Colombia: the relationship between sociodemographic and psychological variables and level of knowledge’, which received approval from the Universidad Libre institutional ethics committee on September 20, 2020. The research project is registered in the Scientific Research Department of the National Autonomous University of Honduras (PI-380-DICIHT). The Declaration of Helsinki’s ethical requirements (1964, amended most recently in 2013) were strictly followed. As a requirement to participate in the study, all participants signed a free and informed consent form. This form provided extensive information about the goal of the study, the methodology used and the rights of the subjects. Participation was completely voluntary and could be discontinued without any consequences.

### Data analysis

Data analysis was carried out in two stages. The first stage entailed generating descriptive statistics to summarize the sociodemographic profile of the sample, along with the outcomes of HPLP-II and DASS-21. Qualitative variables are displayed using frequency distributions and percentages. Measures of central tendency, such as mean, standard deviation, histogram, minimum and maximum values, represent the quantitative variables.

The second stage involved a structural equation analysis (SEM) to assess the empirical feasibility of the suggested theoretical model. The proposed model consisted of 6 observable sociodemographic variables (polychotomous variables were dichotomized), 2 second-level latent variables, 9 first-level latent variables and 73 items (according to the factor structure described for HPLP-II and DASS-21). A confirmatory factor analysis was conducted on the measurement models prior to SEM (this was described in *Measurements* section). The weighted least square mean and variance adjusted estimator was employed due to the categorical and ordinal characteristics of the data (Schumacker and Lomax, 2015).

Adjustment of measurement and structural models were assessed by employing the CFI, the Tucker–Lewis index (TLI) and the RMSEA. CFI and TLI values more than 0.90 and RMSEA values less than or equal to 0.08 are considered as evidence of satisfactory model fit (Schumacker and Lomax, 2015). Data were analyzed using R software, version 4.3.1. The descriptive statistics were generated through skimr package, version 2.1.5 (Waring *et al*., 2022), and the SEM was performed using semTools package, version 0.5-6 (Jorgensen *et al*., 2022) and lavaan version 0.6-16 (Rosseel *et al*., 2023).

## RESULTS

### Descriptive analysis

The study sample consisted of 4203 students, which accounted for 15.14% of the entire population. Table 1 displays the sociodemographic characteristics of the sample. Most of the students identified as women, accounting for 65.48% of the total. The mean age of the entire sample was 21 years (SD = 3 years). Almost half of the study participants (48.01%) were in the age range of 20–24 years. More than two-fifths of participants (42.52%) were enrolled in economic sciences. More than three-fifths (68.36%) reported having a low monthly family income. Furthermore, over one-fifth of the participants (21.20%) engage in studying and working and 13.97% reported having pre-existing medical conditions.

**Table 1:** Sociodemographic characteristics of the study sample

Variable	*N*	%
**Year of enrollment**		
2021	1,740	41.40
2022	1,600	38.07
2023	863	20.53
**Gender**		
Women	2,752	65.48
Men	1,408	33.50
Other/ Non-binary	43	1.03
**Age**		
<20 years	1,777	42.28
20–24 years	2,018	48.01
25–29 years	275	6.54
≥30 years	133	3.17
**Field of knowledge**		
Biological/Health	760	18.08
Economic Sciences	1,787	42.52
Engineering/Physics-Mathematics	728	17.32
Social Sciences/Humanities	850	20.22
Missing	78	1.86
**Monthly family income**		
Low	2,873	68.36
Medium	1,240	29.50
High	90	2.14
**Current occupation**		
Just study	3,312	78.80
Study and work	891	21.20
**Pre-existing medical conditions**		
No	3,616	86.03
Yes	587	13.97

Table 2 displays the descriptive data pertaining to the specific dimensions of the HPLP-II and DASS-21. Concerning the HPLP-II, the dimension of spiritual growth has the greatest average score (X̄ = 24.57), while physical activity has the lowest score (X̄ = 16.23). When examining the possible values of the DASS-21 dimensions, it is observed that depression, anxiety and stress exhibit scores that are around the minimum threshold. This indicates a minimal occurrence of symptoms in all three dimensions.

**Table 2:** Scores of the dimensions of HPLP-II and DASS-21

Scales	Mean	SD	Min.	Max.	Histogram	Skewness	Kurtosis
**HPLP-II**	117.45	23.41	52	208	▁▇▇▂▁	0.45	0.60
Health responsibility	17.85	4.96	9	36	▆▇▅▁▁	0.62	0.42
Physical activity	16.23	5.43	8	32	▆▇▅▂▁	0.55	−0.23
Nutrition	19.72	4.50	9	36	▂▇▇▂▁	0.49	0.56
Spiritual growth	24.57	5.53	9	36	▁▃▇▆▃	−0.15	−0.31
Interpersonal relationships	21.65	4.79	9	36	▁▅▇▃▁	0.23	−0.01
Stress management	17.44	4.02	8	32	▂▇▆▂▁	0.61	0.62
**DASS-21**	20.06	14.16	0	63	▇▇▃▂▁	0.72	−0.16
Depression	6.09	5.56	0	21	▇▃▂▂▁	0.87	−0.14
Anxiety	5.36	4.92	0	21	▇▃▂▁▁	0.96	0.24
Stress	7.13	5.22	0	21	▇▆▅▂▁	0.51	−0.46

### Multivariate analysis

**Fig. 2:**
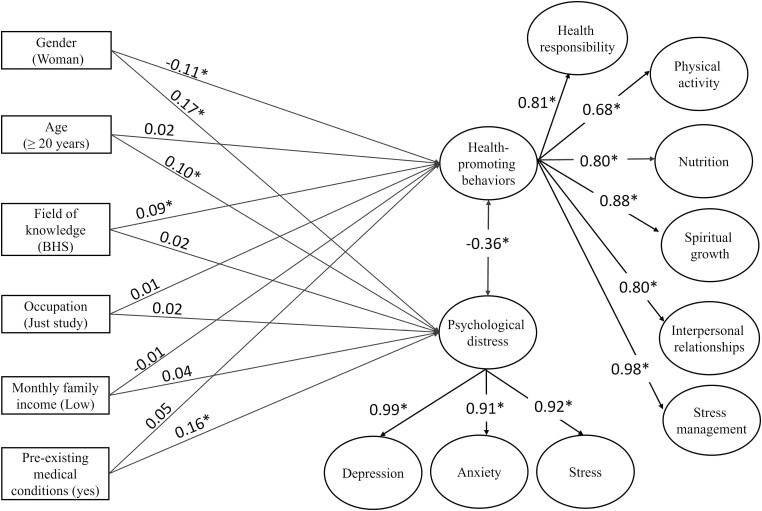
Regressions, correlations and factor loadings of the tested model. Notes: The text in parentheses for each observable variable indicates the reference category; BHS = biological and health sciences; * = *p* < 0.001.

In the structural equation model analysis, all sociodemographic variables were transformed into dummy variables. The predictive model showed a good data fit (*X*^2^ = 46 371.72; *df* = 2971; *p* < 0.001; CFI = 0.951; TLI = 0.957; RMSEA = 0.067 [90% CI = 0.067–0.068]). Figure 2 displays the regressions, correlations and factor loadings of the model. The general factor of health-promoting behaviors exhibited factor loadings ranging from 0.68 to 0.98 for each of the six specific dimensions examined. Psychological distress reflects factor loadings ranging from 0.91 to 0.96 for each of the three specific factors.

Results indicate that only gender and field of knowledge have a statistically significant effect on the prediction of health-promoting behaviors. More precisely, being a woman shows a negative regression (β = −0.11 [95% CI = −0.14, −0.08]; *p* < 0.001), while being enrolled in the field of biological and health sciences depicts a positive regression (β = 0.09 [95% CI = 0.06, 0.13]; *p* < 0.001), although both variables have small effects. Age, occupation, monthly family income and pre-existing medical conditions did not show statistically significant regressions over health-promoting behaviors (*p* > 0.05).

On the other hand, gender, age and pre-existing medical conditions explain psychological distress. Being a woman (β = 0.17 [95% CI = 0.14, 0.21]; *p* < 0.001), aged ≥ 20 years (β = 0.10 [95% CI = 0.06, 0.14]; *p* < 0.001) and having pre-existing medical conditions (β = 0.16 [95% CI = 0.12, 0.19]; *p* < 0.001) were positively associated with psychological distress. The effect sizes of these predictions are considered small. Field of knowledge, occupation, monthly family income and pre-existing medical conditions did not depict statistically significant regressions on psychological distress (*p* > 0.05). Finally, health-promoting behavior and emotional distress showed a statistically significant negative correlation of −0.36 (95% CI = −0.39, −0.32; *p* < 0.001).

## DISCUSSION

We assessed an explanatory model about the relationship among sociodemographic factors, health-promoting lifestyle behaviors and psychological distress in Honduran university students. We conducted univariate and multivariate analyses. The multivariate model demonstrated a good fit to the data.

Our findings indicate that the average score of students on health-promoting lifestyles (117.45 ± 23.41) aligns with the results of prior studies (Alzahrani *et al*., 2019; Safaie *et al*., 2020). Students showed higher scores in the dimension of spiritual growth, which is consistent with the results of some studies in Latin America (Ulla Diez and Perez-Fortis, 2010; Zambrano Bermeo *et al*., 2023). Similarly, recent studies in Saudi Arabia and India reveal higher scores in the spiritual growth dimension (Alzahrani *et al*., 2019; Sahu *et al*., 2020; Al-Momani, 2021). One component of a healthy life is attending to spiritual needs, which might reduce harmful behaviors. A recent study in Iran demonstrated that spiritual health had a positive relationship with the adoption of health-promoting behaviors (Rafat *et al*., 2021).

The lowest score was observed in the dimension of physical activity, which is similar to findings in university students in Colombia and India (Sahu *et al*., 2020; Zambrano Bermeo *et al*., 2023). A recent literature review reports that university students exhibit moderate levels of physical activity (Kljajević *et al*., 2021). Even though universities generally offer an ideal environment for promoting physical activity, our results indicate that lower scores in this dimension may be influenced by various factors, namely, cultural and socio-educational differences.

The multivariate tested model showed that being a woman could be considered a risk factor related to a health-promoting lifestyle (β = −0.11; *p* < 0.001). This outcome aligns with the existing body of research that suggests that health promotion profiles differed by gender, with women particularly having lower scores (Ulla Diez and Perez-Fortis, 2010; Alzahrani *et al*., 2019; Ghanim *et al*., 2021). Previous research reported that women scored higher in interpersonal relationships (Alzahrani *et al*., 2019), whereas our research contradicts this by demonstrating that men score higher in this area (*p* ≤ 0.001; *d* = −0.142). Examining cultural factors and individual differences variables may be required to further understand this association.

The multivariate analysis demonstrated that being enrolled in biological and health sciences serves as a protective factor for fostering a health-promoting lifestyle (β = 0.09; *p* < 0.001). This finding aligns with a recent study conducted in Saudi Arabia, which revealed that university students majoring in healthcare demonstrated higher scores in health responsibility and physical activity, compared to students in other fields (Almutairi *et al*., 2018).

Upon evaluating the psychological distress of students, the average general score of DASS-21 was 20.06 ± 14.16. Regarding specific factors, our research points out lower scores of anxiety (5.36 ± 4.92) and depression (6.09 ± 5.56), and slightly higher scores of stress (7.13 ± 5.22) when compared to the scores reported in recent studies (Slonim *et al*., 2015; Ramón-Arbués *et al*., 2020). Overall, our study sample exhibited minimal psychological distress symptoms. However, due to the screening nature of the DASS-21, these results must be confirmed through comprehensive clinical evaluations.

Our findings showed that being a woman (β = 0.17; *p* < 0.001), aged ≥ 20 years (β = 0.10; *p* < 0.001) and having pre-existing medical conditions (β = 0.16; *p* < 0.001) were predictors of psychological distress. These results align with a study conducted in China, which showed that women achieved notably higher anxiety levels than men during their first and second years of university education (Gao *et al*., 2020). Similarly, a study conducted in Ethiopia demonstrated that being a woman and a first-year student, among other factors, were significantly associated with psychological distress (Tesfahunegn and Gebremariam, 2019).

As confirmed in the multivariate model, age was a statistically significant predictor for psychological distress. Our finding corroborated previous studies conducted in Malaysia (Shamsuddin *et al*., 2013) and China (Cheung *et al*., 2020) which found higher scores of depression, anxiety and stress among students over the age of 20. In addition, pre-existing medical conditions statistically also explain psychological distress. Recent studies show that pre-existing conditions and poor perceived health status were associated with increased risk for moderate to severe depressive and anxiety symptoms (Sayeed *et al*., 2020; Buneviciene *et al*., 2022). Similarly, a study conducted with German students showed that those with pre-existing health conditions had an up to three times higher chance of reporting depressive symptoms (Heumann *et al*., 2023).

We found a statistically significant inverse correlation between health-promoting behavior and psychological distress. This result is consistent with previous literature (Slonim *et al*., 2015; Safaie *et al*., 2020). Adopting healthier lifestyle behaviors appears to have the potential to enhance the mental well-being of students.

Despite its contributions, we need to point out some limitations and perspectives for future studies related to this research. The sample, despite its size and coming from the largest university in Honduras, was selected using a non-probabilistic convenience sampling procedure. Therefore, it is not viable to generalize based on the findings. In addition, the cross-sectional design of this study focused on examining the association between sociodemographic factors, health-promoting lifestyle and psychological distress. Therefore, causal relationships cannot be established. Subsequent research utilizing experimental and longitudinal designs may identify the mechanisms that link the outcome variables and ascertain whether interventions targeting the enhancement of a health-promoting lifestyle would lead to a reduction in psychological distress. This study exclusively focused on first-year students, which restricts the analysis of how the students’ tenure and paths at the university impact the outcome variables. Conducting longitudinal studies would be beneficial to investigate the pattern of change during university life. Another limitation is the reliance on self-report measures, which may yield social desirability responses. To overcome this limitation, future research should employ mixed approaches to gain a more comprehensive understanding of lifestyle-promoting behaviors and psychological distress.

The findings of this study enable the recognition of protective and risk factors of healthy lifestyle behavior and psychological distress in Honduran university students. To the best of our knowledge, in the Honduran context, this is the first study that integrates the analyzed variables into a multivariate structural equation model. This analysis can be leveraged to improve the implementation of both curricular and extracurricular interventions. To develop more targeted and effective interventions, it is important to prioritize sociodemographic groups that have shown statistically significant associations with the outcome variables of this study. Simultaneously, public health authorities and relevant sectors must implement health education and promotion initiatives aimed at the younger demographic. These endeavors will impact not only the immediate health of young individuals but also their long-term well-being and contribute to the overall well-being of the community.

## CONCLUSION

This study evaluated the relationship between sociodemographic factors with healthy lifestyles and psychological distress. The multivariate model showed that a health-promoting lifestyle was significantly influenced by gender and field of knowledge. On the other hand, gender, age and clinical pre-existing medical conditions depicted a significant statistical effect on psychological distress. There was a statistically significant inverse correlation between health-promoting behavior and psychological distress. It is relevant to prioritize future intervention studies targeting sociodemographic groups at risk, with a specific focus on promoting healthy lifestyles and mental well-being.

## Data Availability

The data underlying this article will be shared on reasonable request to the corresponding author.
